# Linking Self-Construal to Creativity: The Role of Approach Motivation and Cognitive Flexibility

**DOI:** 10.3389/fpsyg.2018.01929

**Published:** 2018-10-10

**Authors:** Yan Shao, Bernard A. Nijstad, Susanne Täuber

**Affiliations:** Department of Human Resource Management and Organizational Behavior, Faculty of Economics and Business, University of Groningen, Groningen, Netherlands

**Keywords:** self-construal, creativity, approach motivation, avoidance motivation, cognitive flexibility, cognitive persistence

## Abstract

While some evidence has linked the way individuals define themselves in relation to others (independent versus interdependent self-construal) to creativity, little is known about the underlying mechanism in explaining why and how self-construal influences creativity. Integrating approach-avoidance motivation theory and the dual pathway to creativity model, this research focuses on the motivational and cognitive mechanisms that transfer the effects of self-construal on creativity. Specifically, we expect that independent self-construal is a driver of creativity because it facilitates individuals’ approach motivation, which in turn increases flexible information processing. To test the three-stage mediation model, one experiment and one survey study were conducted. In Study 1, in a sample of 231 Dutch students, self-construal was manipulated by a story-writing task; approach-avoidance motivation, cognitive flexibility, and creativity were measured. In Study 2, self-construal, approach (and avoidance) motivation, cognitive flexibility, and creativity were all measured in a second sample of Dutch students (*N* = 146). The results of two studies supported the three-stage mediation model, showing that approach motivation and cognitive flexibility together mediated the effects of self-construal on creativity. Limitations and implications for future research are discussed.

## Introduction

Since the intriguing publication of Markus and Kitayama (1991) on self-construal, research concerning the implications of individuals’ self-construal on cognition, emotion, and motivation has grown rapidly (see Cross et al., 2011). Self-construal refers to how individuals see themselves in relation to others. Individuals differ in the extent to which they see themselves as autonomous, distinct and unique (independent self-construal) versus as dependent and integral part of larger social groups (interdependent self-construal; Markus and Kitayama, 1991; Kitayama et al., 1997; Gardner et al., 1999).

One important consequence of self-construal is that individuals with different self-construals vary in creativity, defined as generating novel and potentially useful ideas (Amabile, 1983). Some studies have provided preliminary evidence showing that individuals high in independent self-construal relative to those low in independent self-construal or high in interdependent self-construal are more divergent and creative in their thinking (Ng, 2003; Goncalo and Staw, 2006; Wiekens and Stapel, 2008; Jin et al., 2016; Wang and Wang, 2016). However, little is known about the mechanisms underlying the linkage between self-construal and creativity. As suggested by motivated information processing theory that to be creative in generating ideas, individuals need to have a desire to do so (Kunda, 1990; see also Caruso et al., 2006), in the present research, we propose a motivational and cognitive mechanism in explaining the influence of self-construal on creativity by integrating approach-avoidance motivation theory (Elliot and Thrash, 2002; Carver, 2006; Elliot, 2006) and the dual pathway to creativity model (De Dreu et al., 2008; Nijstad et al., 2010).

As a fundamental psychological concept, approach-avoidance motivation has received considerable attention in the study of human behavior (Elliot and Thrash, 2002; Carver, 2006; Elliot, 2006). Approach motivation is conceptualized as the invigoration by or the direction of behaviors toward positive stimuli, whereas avoidance motivation refers to the instigation by or the direction of behaviors away from negative stimuli (Roskes et al., 2013). We suggest that because individuals with high independent self-construal have a tendency to distinguish themselves from others, they are more likely to pursue and obtain positive outcomes that may establish their uniqueness. In contrast, because individuals with high interdependent self-construal emphasize fitting in and harmony, they are motivated to avoid negative outcomes that may disconfirm their relationship with others. Thus, independent self-construal can be linked to approach motivation whereas interdependent self-construal is related to avoidance motivation.

According to the dual pathway to creativity model (De Dreu et al., 2008; Nijstad et al., 2010), creativity can be achieved through either enhanced cognitive flexibility (the use of many and broad cognitive categories or perspectives; Amabile, 1983) or cognitive persistence (the generation of ideas in a few cognitive categories or perspectives; Dietrich, 2004). Personal traits or contextual variables may affect creativity either through the flexibility pathway, the persistence pathway, or both (Nijstad et al., 2010). Research has suggested and shown that when approach motivation is activated, creativity can be achieved through the flexibility pathway, while when avoidance motivation is activated, creativity is achieved though systematic, persistent processing, but only under certain conditions (see Nijstad et al., 2010; Baas et al., 2013).

**FIGURE 1:**
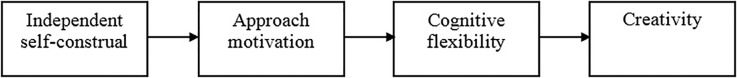
Linking self-construal to creativity: A three-stage mediation model.

Integrating the above insights leads us to propose that independent self-construal is linked to creativity because it is associated with approach motivation, which further promotes cognitive flexibility. Although there are indications that interdependent self-construal is associated with avoidance motivation, the link between avoidance motivation and cognitive persistence is often weak or even negative and depends on additional moderators (e.g., fulfillment of goals; Friedman and Förster, 2002; Baas et al., 2011). Thus, we do not formulate explicit hypothesis about the effects of interdependent self-construal on creativity through avoidance motivation and persistence. The conceptual model is shown in **Figure 1**.

To test the three-stage mediation model, two studies were conducted. First, a laboratory experiment was conducted, in which we manipulated self-construal using a story-writing task and measured approach motivation, cognitive flexibility, and creativity. The experiment enabled us to establish the causal effect of self-construal on approach motivation, cognitive flexibility, and creativity. Second, a survey study was conducted to replicate the lab findings of Study 1 in a Dutch sample of students. With the two complimentary studies, we are able to examine the role of motivation and cognitive flexibility in explaining the effects of self-construal on creativity.

## Theory and Conceptual Model

### Self-Construal and Creativity

Self-construal theory is built on the basic assumption that individuals differ in the way they define and make meaning of themselves in relation to others. Two distinguishable self-construals were first suggested by Markus and Kitayama (1991). Independent self-construal (InSC) refers to the conception of the self as an autonomous, independent unity while interdependent self-construal (InterSC) is defined as the extent to which an individual sees the self as part of an encompassing social relationship (Markus and Kitayama, 1991; Kitayama et al., 1997; Gardner et al., 1999). Although the concept of self-construal was initially used to explain cross-cultural differences in individuals’ representation of self, accumulated research has suggested that individuals within each culture vary in chronic self-construal, and are able to see themselves as more or less independent (or interdependent) according to certain situational cues (Gardner et al., 1999; Lee et al., 2000).

The link between self-construal and creativity has received some preliminary support. At the individual level, based on a sample of 158 white undergraduates from Australia and 186 Chinese undergraduates from Singapore, Ng (2003) study found that independent self-construal had a positive relationship with creative behavior as measured by the Torrance Test of Creative Thinking (TTCT; Torrance, 1972), while interdependent self-construal was negatively related to creativity. Besides, Wiekens and Stapel (2008) demonstrated that the activation of an independent self-construal led to a higher motivation to be independent/different and higher idea generation performance, while the activation of an interdependent self-construal led to a higher motivation to be accepted/to conform and lower idea generation performance.

More recently, Bechtoldt et al. (2010) found that individuals with a Korean background had the default tendency to focus on appropriateness, whereas those with a Dutch background had the default tendency to focus on originality. Given that Korean and Dutch backgrounds are associated with high interdependent self-construal and high independent self-construal, respectively, this research provides indirect support for the relationship between self-construal and creativity. Moreover, based on a sample of junior school students in China, Wang and Wang (2016) found that independent self-construal is more positively associated with self-reported creativity than interdependent self-construal.

At the group level, Goncalo and Staw (2006) found that groups holding individualistic values were more creative than groups holding collectivistic values, especially when originality of responses was emphasized. Although individualism-collectivism is theoretically different from self-construal, research has argued that cultural contexts with different values typically promote the development of one or the other self-construal more strongly (Markus and Kitayama, 1991; Cross et al., 2011). Taken together, these studies provide converging evidence that self-construal is an important antecedent of creativity, and that independent (rather than interdependent) self-construal is a diver of the production of novel, original ideas.

### Self-Construal, Approach-Avoidance Motivation, and Creativity

Motivated information processing theory suggests that to be creative in generating ideas, individuals need to have a desire to do so (Kunda, 1990). We propose that self-construal can influence creativity because it affects motivations that facilitate creativity. Approach-avoidance motivation theory distinguishes between motivation systems that focus on approach and avoidance goals and goal pursuit strategies (Elliot and Thrash, 2002; Carver, 2006; Elliot, 2006). Approach and avoidance motivation can be viewed either as stable personal differences, or as situational variables that can be temporarily activated (Elliot, 2006; Gable and Harmon-Jones, 2008). Approach motivation is conceptualized as the invigoration by or the direction of behaviors toward positive stimuli or possibilities, whereas avoidance motivation refers to the instigation by or the direction of behaviors away from negative stimuli or threats (Roskes et al., 2013).

#### Self-Construal and Approach-Avoidance Motivation

The differences in self-construal have consequences for individuals’ goal pursuits. Individuals high in independent self-construal primarily aim to enhance self-esteem and to be distinct in a positive way. Individuals high in interdependent self-construal generally attempt to defer, to be similar to others and to maintain harmony in social settings (Markus and Kitayama, 1991). Because individuals with discrete self-construals pursue different goals, we predict a relation between self-construal and motivational orientation (approach vs. avoidance).

There are at least two reasons why self-construal is related to approach-avoidance motivation. First, approach motivation guides people’s attention and behavior toward pursuing positive events such as achievement, success, and accomplishment (Elliot and Thrash, 2002), which helps to satisfy individuals’ goal to positively distinguish themselves from others. In contrast, avoidance motivation focuses individuals’ attention and effort on staying away from negative events such as failures, conflicts and mistakes (Elliot and Thrash, 2002), which helps to satisfy individuals’ goal to keep harmony and better fit in in social relationships. Following this reasoning, we propose higher independent self-construal is associated with higher approach motivation, whereas higher interdependent self-construal is linked to higher avoidance motivation. Empirical research has provided some evidence for this argument. For instance, Lee et al. (2000) have demonstrated that individuals high in independent self-construal, primed with independent situations, or with a Western cultural background emphasized approach-related information (achieving success) and showed more affective responses (happiness) associated with approach motivation. In contrast, individuals high in interdependent self-construal, primed with interdependent situations, or with an Eastern cultural background emphasized avoidance-related information (avoiding failure) and showed more affective responses (anxiety) associated with avoidance motivation.

Second, some cross-cultural studies have provided insights into the relationship between self-construal and approach-avoidance motivation. For instance, Elliot et al. (2001) showed that compared with non-Asian Americans, Asian Americans had more avoidance goals and compared with respondents from United States, those from South Korea adopted more avoidance goals (Elliot et al., 2001). In a similar vein, Lockwood et al. (2005) found that individuals with a collectivistic cultural background were more likely to be motivated by negative role models than individuals with an individualistic cultural background. In contrast, positive role models were more motivating for individuals from individualistic cultures rather than for those from collectivistic cultures. Given that individualistic cultures foster a dominant independent self-construal while collectivistic cultures nurture a dominant interdependent self-construal (Markus and Kitayama, 1991), we propose that independent self-construal is linked to approach motivation and interdependent self-construal is associated with avoidance motivation.

#### Approach-Avoidance Motivation and Creativity

Approach-avoidance motivation is associated with creativity because different motivations affect cognitive processing. According to cognitive tuning theory (Schwarz and Bless, 1991), when approach motivation is activated, individuals tend to evaluate the environment as benign. As a consequence, they are more likely to take risks and adopt a relatively heuristic processing style, which in turn enhances creativity. In contrast, when avoidance motivation is activated, individuals tend to judge the environment as problematic and they are more likely to adopt a relatively risk-averse, systematic, and perseverant processing style, which in turn undermines creativity.

A number of studies have supported the link between approach-avoidance motivation and creativity. For instance, Friedman and Förster (2002) demonstrated that bodily cues like arm flexor (associated with approach motivation) relative to arm extensor contraction (associated with avoidance motivation) led to a “riskier,” more heuristic processing style, which in turn boosted creativity in both a problem solving task and idea generation task. Relatedly, Friedman and Forster (2001) showed that cues associated with motivation of pursing idealized goals relative to cues associated with preventing negative outcomes resulted in higher creativity, because the motivation for achieving idealized goals triggered a riskier, explorative processing style than the motivation for preventing negative outcomes. This pattern also held when motivations were measured with individual differences. A more recent study by Roskes et al. (2012) showed that approach motivation generally led to higher creativity compared with avoidance motivation. Avoidance motivated individuals were as creative as approach motivated individuals only when participants were provided with extra motivations that could compensate their effortful processing style (Roskes et al., 2012). In general, we expect that approach motivation has a positive effect on creativity while avoidance motivation might have a negative effect on creativity.

#### Approach-Avoidance Motivation, Cognitive Flexibility, and Creativity

The dual pathway to creativity model (De Dreu et al., 2008; Nijstad et al., 2010) suggests that creativity can be achieved through either enhanced cognitive flexibility (the use of many broad cognitive categories or perspectives: Amabile, 1983) or cognitive persistence (the generation of ideas in a few cognitive categories or perspectives: Dietrich, 2004) and that personal traits or contextual variables may affect creativity either through the flexibility pathway, the persistence pathway, or both. Approach-avoidance motivation has been shown to influence creativity through affecting the pathway individuals adopt. For instance, De Dreu et al. (2011) found that when situations facilitated global, flexible processing, approach motivation potentiated creativity. However, when situations facilitated local, bottom-to-up processing, approach motivation led to lower creativity. This research demonstrated that flexible processing plays an important role in the relationship between approach motivation and creativity. What is more, it has been argued and shown that approach motivation generally boosts creativity because it associates with enhanced activation and cognitive flexibility (Baas et al., 2011).

Research evidence is less consistent about the relationship between avoidance motivation and creativity. Some findings suggested that avoidance motivation promotes creativity and other findings showed no or even negative effects (Friedman and Forster, 2001, Friedman and Förster, 2002; De Dreu et al., 2008). Although avoidance motivation has the potential to boost creativity through persistent processing, research has suggested that avoidance motivation leads to enhanced persistence only when the goals or moods associated with avoidance motivation are activated (Baas et al., 2011) or extra motivation is provided (Roskes et al., 2012). Furthermore, a meta-analysis revealed that creativity is facilitated most by positive activating mood states that are associated with approach motivation (e.g., happiness), rather than moods associated with avoidance motivation (e.g., relaxed, anxious; Baas et al., 2008).

Based on the above arguments and evidence, we expect that approach motivation boosts creativity because it associates with enhanced cognitive flexibility. Given the inconsistent evidence about the link between avoidance motivation, persistence and creativity, we do not have clear expectations about their relationships.

### Self-Construal, Approach-Avoidance Motivation, Cognitive Flexibility, and Creativity

We thus propose that approach motivation plays an important role in transferring the effects of independent self-construal on creativity because it increases cognitive flexibility. Specifically, we propose that individuals high in independent self-construal are more creative as they generally hold higher approach motivation, and this motivation facilitates creativity through enhanced cognitive flexibility, compared with individuals low in independent self-construal. Although we expect that interdependent self-construal is associated with avoidance motivation, according to past research, the relationship between avoidance motivation, persistence, and creativity is difficult to predict without specifying contextual conditions. We thus do not formulate specific hypothesis about the interdependent self-construal-avoidance motivation-persistence-creativity link. Our hypothesis is the following:

Hypothesis 1. Independent self-construal impacts creativity through approach motivation and cognitive flexibility.

## Study 1

### Method

Study 1 was designed to examine whether independent self-construal has a causal effect on creativity through approach motivation and cognitive flexibility. We expected that priming independent self-construal (relative to interdependent self-construal) will temporarily increase individuals’ state approach motivation, which in turn promotes creative performance through enhanced cognitive flexibility. To achieve this goal, we manipulated self-construal using a story-writing task, and measured cognitive flexibility and creative performance with an idea generation task. State approach (and avoidance) motivation were measured with a five-item scale.

#### Sample and Participants

A total of 266 Dutch students (age *M* = 20.65, *SD* = 2.67; 94 women, 168 men, and 4 missing) participated the study for 4 euros or course credits. We randomly assigned all participants to either an interdependent self-construal or independent self-construal condition. In both conditions, participants completed some scales and performed an idea generation task. The study immediately followed another (unrelated) study, and the total session lasted for about 1 h and 15 min.

#### Manipulation and Procedure

Upon arrival in the laboratory, each participant was seated in front of a computer with keyboard. All instructions and measures were given on the computer. Participants were told that the session consisted of several separate parts. Firstly, all participants were asked to finish some personality questionnaires. After that, participants were instructed to perform a story-writing task for 5 min. This was the manipulation of self-construal, which was adopted from Trafimow et al. (1991). In the independent self-construal condition, participants were instructed to think about and write down what makes them different from their family and friends and what they expect themselves to do. In the interdependent self-construal condition, participants were asked to think about and write down what they have in common with their family and friends and what their family and friends expect them to do. Following that, the idea generation task was administered. Participants were instructed to think about and write down as many different and creative uses of a newspaper as possible for 6 min, and the ideas generated had to be neither typical nor virtually impossible. After that, we measured participants’ state approach and avoidance motivation. Subsequently, we collected demographical information, thanked and debriefed all participants.

#### Measures: State Approach/Avoidance Motivation

We measured state motivation using five items on a seven-point Likert scale (1 = not at all, 7 = very much). Items of state approach motivation were “In the problem solving task, I enthusiastically embraced all opportunities to generate solutions” and “In the problem solving task, I was eager to use all possible ways to find solutions or ideas” (*r* = 0.68, *M* = 4.49, *SD* = 1.26). Sample items of state avoidance motivation included “In the problem solving task, I was concerned with making mistakes” and “In the problem solving task, I was cautious about going down the wrong way” (Cronbach’s α = 0.75, *M* = 3.10, *SD* = 1.24). As previous research has shown that avoidance motivation can affect creative performance (e.g., Roskes et al., 2012), we controlled state avoidance motivation in our analysis.

#### Cognitive Flexibility and Creativity

The responses in the newspaper idea generation task were coded for fluency, flexibility, and originality. *Fluency* is the number of non-redundant ideas generated by each participant. *Flexibility* refers to the number of categories that the ideas can be grouped in. Two independent raters coded a subset of responses (30 ideas) for flexibility. The inter-rater agreement (Cohen’s Kappa) was 0.86. Given the good inter-rater agreement, one rater continued to code all ideas. *Originality* was operationalized as the statistical rarity of a given response in a particular sample of subjects, which serves as the indicator of creativity in the present study. Specifically, following Baas et al. (2011), for each idea an originality score was computed: 1-(percentage participants who generated the same idea/100). The scale thus ranged from 0 (low originality) to 1 (high originality). For each participant, the final originality score was the average originality score across all non-redundant ideas.

### Results

#### Data Screening

Two participants did not complete the experiment, thus having missing values on key variables, and three participants wrote down ideas that were not understandable. We excluded these five participants, resulting in 261 participants in our sample.

#### Manipulation Check

We carefully checked the content of participants’ stories to see whether the manipulation was successful. This examination showed that there were 30 participants who did not follow the manipulation instruction correctly. They either wrote down similarities when instructed to write down differences or wrote down differences when instructed to write down similarities. We excluded these 30 participants, resulting in 231 participants in the final sample.

#### Descriptive Statistics

As we can see from **Table 1**, state approach motivation was significantly higher in the independent self-construal condition (*M* = 4.68) than in the interdependent self-construal condition (*M* = 4.30), *t*(229) = −2.36, *p* < 0.05. However, we did not find direct effects of the manipulation of self-construal on other variables (except a marginal significant effect on fluency). The correlation matrix showed that self-construal was significantly correlated with state approach motivation, and state approach motivation was significantly and positively correlated with fluency, flexibility, and originality. State avoidance motivation was significantly and negatively correlated with flexibility and fluency but not originality. Fluency, flexibility, and originality were significantly correlated (*r* > 0.50).

**Table 1:** Study 1 descriptive statistics and correlations.

	Interdependent self-construal	Independent self-construal	*t*-Test		Correlations				
	***M(SD)***	***M(SD)***	***t(df)***	***p***	**2**	**3**	**4**	**5**	**6**
(1) Self-construal^a^					0.15^∗^	0.10	−0.07	−0.11	−0.03
(2) Approach motivation	4.30(1.20)	4.69(1.29)	−2.36(229)	*p* < 0.05		−0.00	0.18^∗∗^	0.19^∗∗^	0.18^∗∗^
(3) Avoidance motivation	2.98(1.24)	3.24(1.23)	−1.58(229)	ns			−0.25^∗∗^	−0.20^∗∗^	−0.12^†^
(4) Flexibility	5.91(2.27)	5.57(2.46)	1.11(229)	ns				0.86^∗∗^	0.63^∗∗^
(5) Fluency	8.59(3.90)	7.68(4.20)	1.58(229)	*p* < 0.10					0.59^∗∗^
(6) Originality	0.63(0.09)	0.62(0.12)	0.51(229)	ns					

*N = 231. ^a^Interdependent self-construal = 0, Independent self-construal = 1. ^†^p < 0.10; ^∗^p < 0.05; ^∗∗^p < 0.01.*

#### Self-Construal, State Approach Motivation, Cognitive Flexibility, and Originality

To test the three-stage mediation model, we used Model 6 of the PROCESS procedure described by Hayes (2013), which allowed us to test the indirect effect of self-construal on creativity through state approach motivation and cognitive flexibility while controlling for avoidance motivation. We generated 95% bootstrap bias-corrected confidence intervals for the indirect effect on the basis of 5000 bootstrap samples. The indirect effect is significant when the confidence intervals does not include zero. The results are shown in **Table 2**. The results demonstrated that the indirect effect of self-construal on originality through state approach motivation and cognitive flexibility was significant (β = 0.004, BootSE = 0.003, BootLLCI = 0.001, and BootULCI = 0.01). The three-stage mediation model was thus confirmed.

**Table 2:** Study 1 regression results of the three-stage mediation model.

Predictors	Dependent variables			
		
	Approach motivation		Flexibility	Originality
Constant	−0.16		0.08	0.63
Avoidance motivation	−0.02		−0.24^∗∗^	0.00
Self-construal^*a*^	0.31^∗^		−0.15	−0.00
Approach motivation			0.19^∗∗^	0.01
Flexibility				0.08^∗∗^
*R*^2^	0.02^†^		0.10^∗∗^	0.40^∗∗^
	**Effect**	**BootSE**	**BootLLCI**	**BootULCI**
Indirect relation^b^	0.004	0.003	0.001	0.01

*N = 231. ^a^0 = interdependent self-construal, 1 = independent self-construal. ^b^Indirect relation = Self-construal-State approach motivation-Cognitive flexibility-Originality. ^†^p < 0.10; ^∗^p < 0.05; ^∗∗^p < 0.01.*

In sum, the results of Study 1 showed that there is a causal relationship between independent self-construal and creativity through state approach motivation and cognitive flexibility while controlling for avoidance motivation, which further confirmed the importance of self-construal in extending the dual pathway to creativity model. However, direct effects of our self-construal manipulation on creativity measures were not observed.

## Study 2

In Study 1, we found some preliminary evidence to support our conceptual model by priming self-construal in the lab. In the second study, we aimed to replicate the lab findings of Study 1 in a different setting where we measured self-construal as a chronic individual difference.

### Method

#### Participants

146 Dutch students (80 men and 66 women) were recruited to participate in this study. Their average age was 21.14. Results did not change when we included gender and age in the analysis, and we excluded these control variables in the report of the results. We invited the participants to the research lab to finish our survey programmed on a computer. The survey consisted of three parts. In the first part, each participant responded to various psychological scales. Following that, they were asked to perform an idea generation task to measure their cognitive flexibility and creativity. Finally, they answered several demographical questions.

#### Measures: Self-Construal

The self-construal scale (SCS; Singelis, 1994) involved a 12-item independent self-construal and a 12-item interdependent self-construal subscale. Sample items of the independent self-construal subscale were “I prefer to be direct and forthright when dealing with people I’ve just met” and “I enjoy being unique and different from others in many respects.” Sample items of the interdependent self-construal subscale included “I have respect for the authority figures with whom I interact” and “It is important for me to maintain harmony within my group.” Participants were instructed to rate the degree to which they agree or disagree with the statements on a seven-point scale, ranging from 1 (strongly disagree) to 7 (strongly agree). The Cronbach’s alpha was 0.68 for independent self-construal and 0.62 for interdependent self-construal.

#### Behavioral Inhibition System/Behavioral Activation System

The Behavioral Inhibition System/Behavioral Activation System (BIS/BAS) scale (Carver and White, 1994), including a 7-item BIS and a 13-item BAS subscale, was used to measure approach-avoidance motivation. Participants were asked to indicate the extent to which the statements reflect themselves on a seven-point scale, ranging from 1 (not true at all of me) to 7 (very much true of me). Sample items of the BAS subscale included “I go out of my way to get things I want” and “I crave excitement and new sensations.” The Cronbach’s alpha was 0.78 for BAS. Sample items of the BIS subscale included “I worry about making mistakes” and “I have very few fears compared to my friends” (reverse scored). The Cronbach’s alpha was 0.81 for BIS.

#### Cognitive Flexibility and Creativity

The measure of creativity was the Tin Can idea generation task (Baas et al., 2011). In this task, participants were asked to generate as many different creative ways to use a tin can as possible and the ideas generated had to be neither typical nor virtually impossible. The responses were coded for fluency, flexibility and originality. The operationalization of fluency and flexibility was the same as Study 1. Two independent raters coded a subset of responses (30 ideas) for flexibility. The inter-rater agreement (Cohen’s Kappa) was 0.96. Given the good inter-rater agreement, one rater subsequently coded all ideas. *Originality* was operationalized with the same way as Study 1.

#### Control Variables

Because interdependent self-construal and avoidance motivation have been suggested to affect individual creative performance (e.g., Friedman and Forster, 2001; Baas et al., 2011), we included them as covariates when testing the multiple-stage mediation model.

### Results

Descriptive statistics, correlations, and scale reliabilities are presented in **Table 3**.

**Table 3:** Study 2 descriptive statistics and correlations.

Variables	*M*	*SD*	1	2	3	4	5	6	7	8	9
(1) Age	21.13	2.21									
(2) Gender	0.54	0.50	−0.15								
(3) Fluency	8.66	3.92	0.23^∗∗^	−0.10							
(4) Flexibility	5.77	2.63	0.18^∗^	−0.11	0.90^∗∗^						
(5) Originality	0.62	0.12	0.09	0.02	0.66^∗∗^	0.67^∗∗^					
(6) InSC	4.76	0.69	0.10	0.20^∗^	0.23^∗∗^	0.25^∗∗^	0.27^∗∗^	0.68			
(7) InterSC	4.45	0.60	−0.30^∗∗^	−0.11	−0.10	−0.10	−0.14	−0.13	0.62		
(8) BAS	5.20	0.61	0.02	−0.02	0.26^∗∗^	0.27^∗∗^	0.20^∗^	0.43^∗∗^	0.08	0.81	
(9) BIS	4.63	0.99	0.03	−0.25^∗∗^	0.08	0.06	0.12	−0.26^∗∗^	0.16^∗^	0.00	0.78

*N = 146. InSC = independent self-construal; InterSC = interdependent self-construal. Gender: 0 = women; 1 = men. In the correlation matrix, numbers at the diagonal are Cronbach’s α values for measurement scales used in the current study. ^∗^p < 0.05; ^∗∗^p < 0.01.*

#### Confirmatory Factorial Analysis (CFA)

We performed confirmatory factorial analysis (CFA) (Lavaan 0.5-23 R package) to examine the discriminant validity of self-construal and BIS/BAS motivation (see **Table 4**). We compared fit statistics of five alternative models to the baseline model by means of χ^2^-differences, root-mean-square error of approximation (RMSEA), the comparative fit index (CFI), the Tucher-Lewis index (TLI), and the standardized root-mean-square residual (SRMR). To enhance model parsimony, following Ng (2003), we randomly packaged measurement items to a small number of groups. Specifically, we randomly assigned the 12 items to 3 parcels for independent self-construal and interdependent self-construal, respectively. Similarly, we randomly packaged the 13 items to form 3 indicators for BAS and the 7 items to form 3 indicators for BIS. Item parceling has been suggested to enhance model parsimony by reducing the number of indicators and better meet the assumption of maximum likelihood estimation procedure used in the structural equation modeling (Finch and West, 1997).

**Table 4:** Study 2 confirmatory factor analysis.

Factor structure	χ^2^	df	RMSEA	CFI	TLI	SRMR	Δχ^2^(Δdf)
Baseline model: four factors	69.03	48	0.06	0.95	0.94	0.06	
Model1: one factor	312.40	54	0.18	0.43	0.30	0.15	243.37(6)^∗∗∗^
Model2: two factors	167.28	53	0.12	0.75	0.69	0.11	98.26(5)^∗∗∗^
Model3: two factors	266.61	53	0.17	0.53	0.41	0.16	197.58(5)^∗∗∗^
Model4: three factors	100.92	51	0.08	0.90	0.86	0.08	31.89(3)^∗∗∗^
Model5: three factors	137.58	51	0.11	0.81	0.75	0.10	68.55(3)^∗∗∗^
Model6: three factors	225.04	51	0.15	0.62	0.50	0.15	156.02(3)^∗∗∗^
Model7: three factors	102.57	51	0.08	0.89	0.85	0.09	33.54(3)^∗∗∗^
Model8: common latent factor	61.40	44	0.05	0.96	0.94	0.06	7.63(4)

*N = 146. Δχ^2^ and Δdf refer to the differences with the baseline model. Model 1: All variables on one factor; Model 2: Independent self-construal and BAS on one factor while interdependent self-construal and BIS on another factor; Model 3: Independent and interdependent self-construal on one factor while BIS and BAS on another factor; Model 4: Interdependent self-construal and BIS on one factor; Model 5: Independent self-construal and BAS on one factor; Model6: BIS and BAS on one factor; Model7: Interdependent and independent self-construal on one factor. Model8: Adding a latent factor with all of the items as indicators to the baseline model. ^∗∗∗^p < 0.001.*

Results from CFA analysis showed that the hypothesized baseline model (independent self-construal, interdependent self-construal, BIS and BAS) fitted the data well [χ^2^(48) = 69.03, *p* < 0.05; CFI = 0.95, TLI = 0.94, RMSEA = 0.06, RSMR = 0.06]. The four-factor model was significantly better than the one factor model where all indicators loaded on a single factor [Δχ^2^(6) = 243.37, *p* < 0.001], and showed a better fit than all other alternative models. This confirms the discriminant validity of the four constructs.

#### Common Method Bias

Because both self-construal and BIS/BAS motivation were measured using self-reports on a Likert scale, we examined the degree to which common method bias was present in the current study with common latent factor analysis (e.g., Williams et al., 1989; Podsakoff et al., 2003). Compared with the single-factor test (Harman, 1960), the common latent factor approach allows for the consideration of measurement error and does not require the researcher to identify the specific factor responsible for common method effects. This analysis was conducted by adding a latent factor with all of the items as indicators to our four-factor model (see **Table 4**). The paths from the indicators to the common factor were constrained to be equal and the variance of the common factor was constrained to be 1 to make sure the model can be identified (Eichhorn, 2014). The results showed that adding a common method factor did not improve the mode fit significantly [Δχ^2^(2) = 7.63, *p* > 0.10], which suggests that common method bias is not a serious concern in the current study.

#### The Three-Stage Mediation Model

We predicted that independent self-construal has an indirect effect on creativity through approach motivation and cognitive flexibility. The three-stage mediation model was tested using Model 6 of the PROCESS tool described by Hayes (2013). As shown in **Table 5**, after controlling for interdependent self-construal and avoidance motivation, independent self-construal had a significant indirect effect of on creativity through BAS and cognitive flexibility (β = 0.007, BootSE = 0.003, BootLLCI = 0.002, and BootULCI = 0.016), replicating Study 1. The results confirmed that independent self-construal affected creativity through enhanced approach motivation and cognitive flexibility

**Table 5:** Study 2 regression results of the three-stage mediation model.

Predictors	Dependent variables			
	BAS	Flexibility		Originality
Constant	−0.13	0.01		0.61^∗∗^
InterSC	0.11	−0.14		−0.01
BIS	0.09	0.15		0.01^†^
InSC	0.46^∗∗^	0.18^†^		0.02^†^
BAS		0.23^∗^		−0.00
Flexibility				0.07^∗∗^
*R*^2^	0.19^∗∗^	0.11^∗∗^		0.46^∗∗^
Indirect relation	**Indirect effect**	**BootSE**	**BootLLCI**	**BootULCI**
	0.007	0.003	0.002	0.016

*N = 146. InSC = independent self-construal; InterSC = interdependent self-construal. Indirect Relation, Independent self-construal-BAS-Cognitive flexibility-Originality. ^†^p < 0.10; ^∗^p < 0.05; ^∗∗^p < 0.01.*

## General Discussion

Earlier research has highlighted the role of self-construal as an important source of creativity, but the existing literature is fragmented in terms of how and why self-construal is linked to creativity. Our research proposed that approach-avoidance motivation may serve as a motivational mechanism in explaining the effects of self-construal on creativity. Drawing on the dual pathway to creativity model, we further proposed that self-construal affects creativity because it enhances individuals’ approach motivation, which in turn facilitates flexible information processing in ideation.

Our conceptual model was supported in two complementary studies. In Study 1, we found that individuals primed with independent self-construal, relative to those primed with interdependent self-construal, were higher in state approach motivation, and state approach motivation was significantly and positively linked to cognitive flexibility and originality. The mediation analysis showed a significant three-stage indirect effect after controlling for avoidance motivation. In other words, findings of Study 1 supported our hypothesis that self-construal influences creativity through state approach motivation and cognitive flexibility. However, although we found that priming self-construal temporarily enhanced individuals’ approach motivation, we did not find a significant direct effect of self-construal on creativity. One possible reason is that the manipulation was not strong enough to produce a direct effect, because self-construal and creativity are more distally related than self-construal and motivation. In Study 2, we conducted a survey among a Dutch student sample. We found that after controlling for avoidance motivation and interdependent self-construal, approach motivation, and cognitive flexibility together mediated the effects of independent self-construal on creativity, replicating the findings of Study 1.

### Theoretical Implications

The present research takes a step toward uncovering the mechanism underlying the link between self-construal and creativity. Previous studies have begun to identify that independent self-construal is linked to motivation to be independent/different whereas interdependent self-construal induces motivation to be accepted/to conform (Wiekens and Stapel, 2008). However, little research has addressed the possibility that the motivation resulting from self-construal can mediate the effects of self-construal on creativity. Besides, despite that some studies have found a positive link between approach motivation and creativity because of flexibility (e.g., Roskes et al., 2012), little attention has been paid to reveal the sources of approach motivation. Our three-stage mediation model integrated previous fragmented literatures by demonstrating that approach motivation and cognitive flexibility sequentially mediate the relationship between independent self-construal and creativity.

Second, the mediators being tested in the present research have strong implications for uncovering future moderators of the relationship between self-construal and creativity. The present study shows that self-construal influences creativity because of approach motivation and cognitive flexibility. Therefore, we can expect that under some circumstances, the positive relationship between independent self-construal and creativity may not hold because the conditions do not afford approach motivation and/or flexible information processing. For example, past research suggested that approach motivation has a positive link with creativity only if the situation affords flexible and global processing (De Dreu et al., 2011). In a similar vein, we may expect that independent self-construal leads to creativity only if the situation makes approach motivation and/or cognitive flexibility feasible. This study thus encourages future research to investigate contextual factors which moderate the relationship between independent self-construal and creativity.

In addition, this research examined the indirect effect of independent self-construal on creativity with mixed methods. Some past research has either used surveys or laboratory experiments. Our two complementary studies provide consistent support for the three-stage mediation model, which increases confidence about the indirect effect of independent self-construal on creativity. Specifically, we contributed knowledge that both situationally primed self-construal and chronic self-construal are associated with creative performance through approach motivation and cognitive flexibility.

### Practical Implications

The central implication for management practices from this study is the challenge to realize the potential of independent self-construal for creative production. This research provides insights into manageable interventions that can be used to promote individual creativity. For instance, because self-construal is often stable and difficult to change, for employees low in independent self-construal, it might be more effective for managers to provide and emphasize rewards, achievements and train the employees with approach orientated strategies (e.g., the use of intuition) to achieve creativity than to change employee’s self-definition. In addition, creating conditions that facilitate cognitive flexibility is critical to increase employees’ creativity. For instance, research has shown that individuals with activated positive mood (e.g., happy) are more creative than those with deactivated positive mood (e.g., relaxed) because of differences in cognitive flexibility (De Dreu et al., 2008). Therefore, creating a work environment that helps employees be happy is beneficial for cognitive flexibility, which in turn boosts creativity.

### Limitations and Avenues for Future Research

The contributions of the current study should be seen in light of several limitations. First, we only adopted one cognitive manipulation of self-construal. We are not certain whether the effects we observed in our experiment can be generalized to different manipulations such as the word search task (Brewer and Gardner, 1996), Sumerian warrior task (Trafimow et al., 1991) and a different version of story-writing task (Utz, 2004). Second, we used a single measure of creativity in the present research. Although idea generation tasks are widely used to assess creativity, the effect we observed for the idea generation task (divergent thinking task) may not hold for other convergent thinking tasks. For example, Shen et al. (2018) have found that although risk-taking orientation is not significantly related to divergent thinking performance, it has a significant, negative association with convergent thinking performance. Future research is thus encouraged to employ the Remote Associates Test (RAT: Mednick and Mednick, 1967) or other convergent thinking tasks to investigate the effects of self-construal on creativity. Third, our model was tested only in Dutch samples. Future research can address this limitation by testing our model in other cultures.

Also, in the current study we chose to only focus on the link between independent self-construal and originality of ideas. However, we believe that it is equally important to study how interdependent self-construal influences appropriateness or usefulness of ideas. Some evidence has suggested that individuals with different self-construals tend to have different biases toward creativity (e.g., Bechtoldt et al., 2010). Specifically, it seems that people with independent self-construal are motivated to stand out and be original in idea generation, while people with interdependent self-construal are motivated to be similar and generate mainly appropriate and useful ideas. Future study could directly examine this possibility by measuring both originality and appropriateness of ideas. Moreover, if people with different self-construals tend to focus on either originality or appropriateness of creativity, one intriguing question is how individuals can be ambidextrous in creativity by achieving appropriateness and originality simultaneously given that both aspects are important for creativity. Indeed, a few studies have started to investigate the conditions that can foster both appropriateness and originality simultaneously and have shown it is possible for individuals to be ambidextrous in creativity (e.g., Miron-Spektor and Beenen, 2015). In addition, Zhang et al. (2015) have shown that leaders can demonstrate paradoxical behaviors, creating a work environment that fosters employees’ productivity and adaptivity simultaneously. In sum, the current study takes the first step to examine individuals’ bias toward creativity because of their self-construal, and future studies may investigate the question how and why such a bias can be managed to achieve high creativity.

Finally, our second study used a self-report method to measure both independent self-construal and approach motivation. Although the common latent factor analysis showed that the common method bias is unlikely to threaten the validity of our results, future study is encouraged to reduce common method bias by, for example, measuring the two constructs with different methods or from different sources.

## Conclusion

Scholars tend to argue that for individuals to be creative, they need to have the motivation to do so (Kunda, 1990). Although research has demonstrated that self-construal is a predictor of creativity, the mechanism underlying the self-construal and creativity link is unclear. The present study provides empirical evidence for the motivational mechanism, in that it showed that approach motivation plays a role in explaining the influences of independent self-construal on creativity. More importantly, this research showed that approach motivation mediates the self-construal-creativity link because it gives rise to cognitive flexibility. The motivational and cognitive mechanism clearly explains how and why independent self-construal impacts creativity.

## Ethics Statement

This study was carried out in accordance with the recommendations of recommendations of the research ethics guidelines of the Research Ethics Committee of the Faculty of Economics and Business of the University of Groningen with written informed consent from all subjects. All subjects gave written informed consent in accordance with the Declaration of Helsinki. The protocol was approved by the Research Ethics Committee of the Faculty of Economics and Business of the University of Groningen.

## Author Contributions

YS and BN developed the research idea together. Under the supervision of BN, YS collected and analyzed the data for Studies 1 and 2. YS took the lead in drafting the manuscript. BN and ST commented on the draft.

## Conflict of Interest Statement

The authors declare that the research was conducted in the absence of any commercial or financial relationships that could be construed as a potential conflict of interest.
